# Salutatory

**Published:** 1883-05

**Authors:** J. H. Logan


### Editorial.

### SALUTATORY.

It is with some trepidation that I herewith assume editorial charge of the REGISTER. The traditional mantle falls upon me from the shoulders of a young and vigorous predecessor, whose conservative, graceful pen will be missed hereafter in the pages of the REGISTER.

Increasing professional engagements necessitated this [illegible] d I heartily wish for him in his retiracy, under the exacting duties of the present, and the new business relations of which he spoke in the future, all the success which his admirable social qualities deserve and his talents are so capable of maintaining.

And now that I am fairly mustered into service, I may not underrate for a moment the responsibilities and duties that await me. In this age of unsurpassed intellectual activity, the power of the press has developed into proportions never before witnessed in the world's history. It has come to be the directing force in every enterprise, whether literary, political or scientific.

It is a matter of just pride to the profession that in this grand march of improvement the medical press is not lagging in the rear. I venture the assertion that in the whole range of magazine literature, there is none more independent and earnest in tone, none freer from personal bitterness or more pervaded by broad, liberal thought, and characterized with all by a more elegant, scholarly diction than is, at this hour, the medical press of our country and time.

It would not be difficult, were it in order, to show this by numerous examples taken at random from contemporary medical literature of Europe and America.

Hence the first installment of responsibility to him who presumes now to sit as a scribe in the temple of Æsculapius. But there is yet another and heavier installment. The field to be cultivated is an immense one, and both traditionally and in the nature of things beset with difficulties.

Many questions of momentous import are pressing for solution; new generalizations are asking for clearer statement and formulation; numerous claims are to be sifted and properly adjusted; established principles to be defended, popularized and secured. And last, but not least, the entire field is to be often explored and re-explored in the patient search for new material and facts for future generalizations.

In the meantime, scarcely restrained epidemics, and foul diseases no less deadly, but more stealthy and diffused, are scurging the world and filling its homes with sorrow and despair.

In this view of the situation, there is no room for boasting—not a foot-place for presumption. As for myself, I can do nothing better now than to ask of my more experienced brethren of the Æsculapian tripod some degree of indulgence, till, by and by, being inured to the work, I may more efficiently co operate with them in promoting the best interest and true dignity of our noble profession. J. H. LOGAN, M. D.

